# Methyl *trans*-*rac*-2-hexyl-1-oxo-3-(2-pyrid­yl)-1,2,3,4-tetra­hydroisoquinoline-4-carboxyl­ate

**DOI:** 10.1107/S1600536808029048

**Published:** 2008-09-13

**Authors:** Sema Öztürk Yıldırım, Mehmet Akkurt, Meglena I. Kandinska, Milen G. Bogdanov, Orhan Büyükgüngör

**Affiliations:** a Department of Physics, Faculty of Arts and Sciences, Erciyes University, 38039 Kayseri, Turkey; b Faculty of Chemistry, University of Sofia, 1 James Bourchier blvd., 1164 Sofia, Bulgaria; c Department of Physics, Faculty of Arts and Sciences, Ondokuz Mayıs University, 55139 Samsun, Turkey

## Abstract

The title compound, C_22_H_26_N_2_O_3_, was synthesized by esterification of *trans*-*rac*-2-hexyl-1-oxo-3-(2-pyrid­yl)-1,2,3,4-tetra­hydro­isoquinoline-4-carboxylic acid in the presence of H_2_SO_4_ in methanol. The dihedral angle between the benzene and pyridine rings is 84.46 (17)°. The piperidine ring adopts a screw-boat conformation. In the crystal, inversion dimers linked by two C—H⋯O bonds occur.

## Related literature

 For background on potential applications of this family of compounds and the synthesis, see: Kandinska *et al.* (2006 ▶, 2007 ▶). For bond-length data, see: Allen *et al.* (1987 ▶). For puckering parameters, see: Cremer & Pople (1975 ▶).
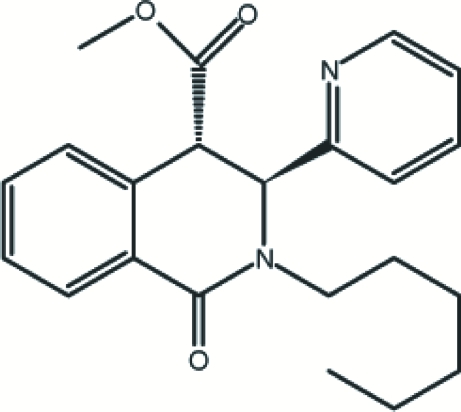


## Experimental

### 

#### Crystal data

C_22_H_26_N_2_O_3_
*M* *_r_* = 366.45
Orthorhombic, 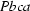

*a* = 8.8404 (2) Å
*b* = 15.6719 (5) Å
*c* = 29.1488 (10) Å
*V* = 4038.5 (2) Å^3^
*Z* = 8
Mo *K*α radiation
μ = 0.08 mm^−1^
*T* = 293 (2) K
0.69 × 0.63 × 0.57 mm

#### Data collection

Stoe IPDS 2 diffractometer
Absorption correction: integration (*X-RED32*; Stoe & Cie, 2002 ▶) *T*_min_ = 0.947, *T*_max_ = 0.956
30845 measured reflections
3735 independent reflections
2647 reflections with *I* > 2σ(*I*)
*R*_int_ = 0.078

#### Refinement

*R*[*F* ^2^ > 2σ(*F* ^2^)] = 0.089
*wR*(*F* ^2^) = 0.279
*S* = 1.07
3735 reflections
238 parameters
2 restraints
H-atom parameters constrained
Δρ_max_ = 0.71 e Å^−3^
Δρ_min_ = −0.58 e Å^−3^

### 

Data collection: *X-AREA* (Stoe & Cie, 2002 ▶); cell refinement: *X-AREA*; data reduction: *X-RED32* (Stoe & Cie, 2002 ▶); program(s) used to solve structure: *SIR97* (Altomare *et al.*, 1999 ▶); program(s) used to refine structure: *SHELXL97* (Sheldrick, 2008 ▶); molecular graphics: *ORTEP-3 for Windows* (Farrugia, 1997 ▶); software used to prepare material for publication: *WinGX* (Farrugia, 1999 ▶).

## Supplementary Material

Crystal structure: contains datablocks global, I. DOI: 10.1107/S1600536808029048/wn2280sup1.cif

Structure factors: contains datablocks I. DOI: 10.1107/S1600536808029048/wn2280Isup2.hkl

Additional supplementary materials:  crystallographic information; 3D view; checkCIF report

## Figures and Tables

**Table 1:** Hydrogen-bond geometry (Å, °)

*D*—H⋯*A*	*D*—H	H⋯*A*	*D*⋯*A*	*D*—H⋯*A*
C6—H6⋯O2^i^	0.93	2.54	3.460 (5)	169

Symmetry code: (i) 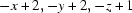.

**Fig. 1.**
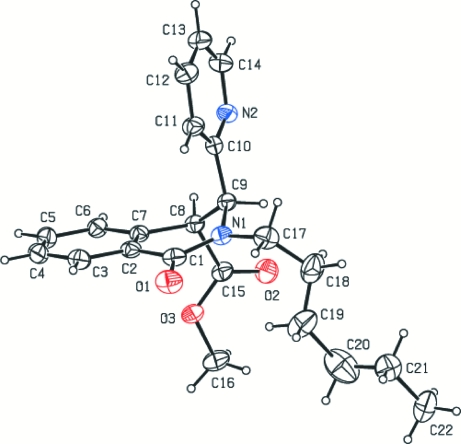
A view of the molecular structure of the title compound, with the atom-numbering scheme and displacement ellipsoids drawn at the 20% probability level. H atoms are represented by spheres of arbitrary radius.

**Fig. 2.**
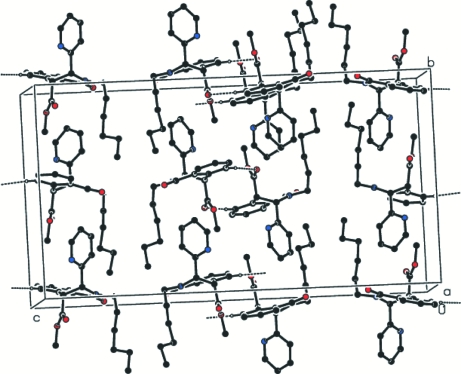
A view of the packing and hydrogen bonding (dashed lines) of the title compound, viewed down the a-axis. H atoms not involved in hydrogen bonding have been omitted for clarity.

## supplementary crystallographic information

### Comment

The title compound was synthesized as part of a research project to find precursors for the production of new tetrahydroquinolone derivatives with potential biological activity (Kandinska *et al.*, 2006; Kandinska *et al.*, 2007).

The molecular structure is shown in Fig.1. The bond lengths and angles are in normal ranges (Allen *et al.*, 1987). The dihedral angle between the benzene and pyridine rings is 84.46 (17) °. The piperidine ring adopts a screw boat conformation and its puckering parameters (Cremer & Pople, 1975) are Q_T_ = 0.465 (3) Å, θ = 114.9 (4)° and φ = 93.7 (4) °.

The crystal structure is stabilized by intermolecular C—H···O hydrogen bonds (Table 1 and Fig. 2).

### Experimental

The title compound was synthesized by esterification of *trans*-*rac*-2-hexyl-1-oxo-3-(pyridin-2-yl)-1,2,3,4-tetrahydroisoquinoline-4-carboxylic acid (3.81 g, 0.011 mol) (Kandinska *et al.*, 2007) in the presence of H_2_SO_4_ (1.7 ml, 0.032 mol) in methanol. After working up the reaction mixture, the title compound crystallized as white crystals from ethyl acetate (yield 3.56 g, 90%; m.p. 357–359 K). Analysis, calculated for C_22_H_26_N_2_O_3_ (366.45): C 72.11, H 7.15%; found: C 72.35, H7.08%. The product was further characterized by ^1^H NMR and IR spectra.

### Refinement

All H atoms were positioned geometrically and allowed to ride on their attached atoms, with C—H distances = 0.93 - 0.97 Å, and with *U*_iso_(H) =1.2 or 1.5*U*_eq_(carrier atom). Atoms C19, C20 and C21 in the hexyl group appear to have unresolved disorder, so atom C21 was refined isotropically and the distances C19—C20 and C20—C21 were restrained by *SHELXL DFIX* instructions to a value of 1.530 Å (Allen *et al.*, 1987). Probably due to the poor crystal quality, the observed and calculated structure factors showed rather large disagreement. Hence, to improve the R factor, 81 reflections were suppressed in the refinement process.

### Crystal data

**Table:**

C_22_H_26_N_2_O_3_	*F*(000) = 1568
*M**_r_* = 366.45	*D*_x_ = 1.205 Mg m^−^^3^
Orthorhombic, *P**b**c**a*	Mo *K*α radiation, λ = 0.71073 Å
Hall symbol: -P 2ac 2ab	Cell parameters from 35910 reflections
*a* = 8.8404 (2) Å	θ = 1.3–26.1°
*b* = 15.6719 (5) Å	µ = 0.08 mm^−^^1^
*c* = 29.1488 (10) Å	*T* = 293 K
*V* = 4038.5 (2) Å^3^	Block, colourless
*Z* = 8	0.69 × 0.63 × 0.57 mm

### Data collection

**Table:**

STOE IPDS 2 diffractometer	3735 independent reflections
Radiation source: sealed X-ray tube, 12 x 0.4 mm long-fine focus	2647 reflections with *I* > 2σ(*I*)
plane graphite	*R*_int_ = 0.078
Detector resolution: 6.67 pixels mm^-1^	θ_max_ = 25.7°, θ_min_ = 1.4°
ω scans	*h* = −10→10
Absorption correction: integration (X-RED32; Stoe & Cie, 2002)	*k* = −18→18
*T*_min_ = 0.947, *T*_max_ = 0.956	*l* = −34→35
30845 measured reflections	

### Refinement

**Table:**

Refinement on *F*^2^	Primary atom site location: structure-invariant direct methods
Least-squares matrix: full	Secondary atom site location: difference Fourier map
*R*[*F*^2^ > 2σ(*F*^2^)] = 0.089	Hydrogen site location: inferred from neighbouring sites
*wR*(*F*^2^) = 0.279	H-atom parameters constrained
*S* = 1.07	*w* = 1/[σ^2^(*F*_o_^2^) + (0.1761*P*)^2^ + 0.794*P*] where *P* = (*F*_o_^2^ + 2*F*_c_^2^)/3
3735 reflections	(Δ/σ)_max_ < 0.001
238 parameters	Δρ_max_ = 0.71 e Å^−^^3^
2 restraints	Δρ_min_ = −0.58 e Å^−^^3^

### Special details

**Table:**

Experimental. Single crystals were obtained by slow evaporation of a solution of the title compound in ethyl acetate at room temperature. IR (CHCl_3_) 1600 cm^-1^ (ArH), 1660 cm^-1^ (C=O), 1740 cm^-1^ (C=O). ^1^HNMR (250 MHz, CDCl_3_) δ (p.p.m.) = 0.83–0.86 (m, 3H, *-C*H*_3_*), 1.18–1.35 (m, 6H, *-C*H*_2_-*), 1.58–1.67 (m,2*H*, *-C*H*_2_-*), 2.81–2.88 (m,1*H*, *N—C*H*_2_^a^*), 3.70 (s, 3H, *-OC*H*_3_*), 4.20–4.28 (m, 1H, *N—C*H*_2_^b^*), 4.42 (s, 1H, *-OOC-C*H), 5.32 (s, 1H, *Pyr-C*H, 6.90–6.98 (m, 1H, *Ph-*H), 7.08–7.19 (m, 2H, *Ph-*H, *Pyr-*H), 7.25–7.30 (m, 2H, *Pyr-*H), 7.36–7.47 (m, 1H, *Ph-*H), 7.87 (dd, 1H, *J* = 2.0 and 10.0 Hz, *Ph-*H),8.08 (dm, 1H,*J* = 4.0 Hz, *Pyr-*H).
Geometry. Bond distances, angles *etc*. have been calculated using the rounded fractional coordinates. All su's are estimated from the variances of the (full) variance-covariance matrix. The cell e.s.d.'s are taken into account in the estimation of distances, angles and torsion angles
Refinement. Refinement on *F*^2^ for ALL reflections except those flagged by the user for potential systematic errors. Weighted *R*-factors *wR* and all goodnesses of fit *S* are based on *F*^2^, conventional *R*-factors *R* are based on *F*, with *F* set to zero for negative *F*^2^. The observed criterion of *F*^2^ > σ(*F*^2^) is used only for calculating -*R*-factor-obs *etc*. and is not relevant to the choice of reflections for refinement. *R*-factors based on *F*^2^ are statistically about twice as large as those based on *F*, and *R*-factors based on ALL data will be even larger.

### Fractional atomic coordinates and isotropic or equivalent isotropic displacement parameters (Å^2^)

**Table:**

	*x*	*y*	*z*	*U*_iso_*/*U*_eq_	
O1	0.6287 (3)	1.0146 (2)	0.67997 (10)	0.0918 (11)	
O2	1.0639 (3)	0.9186 (2)	0.56576 (14)	0.1041 (13)	
O3	0.8206 (3)	0.89875 (15)	0.55212 (11)	0.0811 (10)	
N1	0.8646 (3)	1.03830 (17)	0.65188 (9)	0.0595 (9)	
N2	1.0890 (3)	1.19495 (18)	0.58718 (10)	0.0654 (10)	
C1	0.7102 (4)	1.0356 (2)	0.64749 (13)	0.0661 (11)	
C2	0.6468 (4)	1.05917 (18)	0.60251 (12)	0.0574 (10)	
C3	0.4909 (4)	1.0776 (2)	0.59856 (16)	0.0749 (14)	
C4	0.4295 (4)	1.0982 (2)	0.55687 (17)	0.0791 (14)	
C5	0.5176 (4)	1.1012 (2)	0.51854 (16)	0.0760 (14)	
C6	0.6711 (4)	1.0827 (2)	0.52161 (13)	0.0638 (11)	
C7	0.7356 (3)	1.06194 (18)	0.56354 (11)	0.0534 (9)	
C8	0.9013 (3)	1.04072 (19)	0.56832 (11)	0.0539 (9)	
C9	0.9606 (3)	1.07082 (18)	0.61483 (11)	0.0532 (9)	
C10	0.9825 (3)	1.16633 (19)	0.61558 (11)	0.0536 (9)	
C11	0.9063 (4)	1.2211 (2)	0.64487 (14)	0.0703 (11)	
C12	0.9425 (5)	1.3067 (3)	0.64457 (16)	0.0840 (16)	
C13	1.0519 (6)	1.3364 (3)	0.61556 (17)	0.0883 (16)	
C14	1.1231 (5)	1.2765 (3)	0.58783 (16)	0.0837 (14)	
C15	0.9396 (4)	0.9465 (2)	0.56223 (12)	0.0612 (11)	
C16	0.8489 (6)	0.8081 (3)	0.54473 (19)	0.101 (2)	
C17	0.9382 (5)	1.0220 (3)	0.69588 (14)	0.0853 (16)	
C18	1.0400 (6)	0.9436 (4)	0.6965 (2)	0.118 (3)	
C19	0.9589 (8)	0.8632 (4)	0.6907 (3)	0.131 (3)	
C20	1.0378 (9)	0.7692 (6)	0.6833 (3)	0.169 (4)	
C21	1.1265 (7)	0.7497 (4)	0.7199 (2)	0.1150*	
C22	1.2046 (6)	0.6616 (4)	0.7108 (2)	0.121 (3)	
H3	0.42950	1.07570	0.62450	0.0900*	
H4	0.32670	1.11020	0.55470	0.0950*	
H5	0.47510	1.11560	0.49040	0.0910*	
H6	0.73090	1.08430	0.49540	0.0770*	
H8	0.95590	1.07250	0.54460	0.0650*	
H9	1.06070	1.04510	0.61890	0.0640*	
H11	0.83180	1.20040	0.66450	0.0840*	
H12	0.89240	1.34410	0.66410	0.1010*	
H13	1.07750	1.39390	0.61440	0.1060*	
H14	1.19990	1.29540	0.56850	0.1000*	
H16A	0.75540	0.77980	0.53780	0.1520*	
H16B	0.89210	0.78370	0.57200	0.1520*	
H16C	0.91790	0.80100	0.51960	0.1520*	
H17A	0.86080	1.01510	0.71920	0.1020*	
H17B	0.99820	1.07150	0.70410	0.1020*	
H18A	1.11430	0.94890	0.67220	0.1420*	
H18B	1.09430	0.94210	0.72540	0.1420*	
H19A	0.89210	0.87150	0.66470	0.1570*	
H19B	0.89410	0.85770	0.71740	0.1570*	
H20A	0.95990	0.72610	0.67980	0.2030*	
H20B	1.09850	0.76970	0.65560	0.2030*	
H21A	1.06560	0.74660	0.74750	0.1380*	
H21B	1.20260	0.79350	0.72410	0.1380*	
H22A	1.25770	0.64370	0.73780	0.1810*	
H22B	1.27470	0.66710	0.68580	0.1810*	
H22C	1.12910	0.61990	0.70310	0.1810*	

### Atomic displacement parameters (Å^2^)

**Table:**

	*U*^11^	*U*^22^	*U*^33^	*U*^12^	*U*^13^	*U*^23^
O1	0.0917 (19)	0.100 (2)	0.0837 (19)	−0.0117 (16)	0.0350 (16)	0.0091 (15)
O2	0.0659 (16)	0.0784 (19)	0.168 (3)	0.0230 (14)	0.0148 (18)	−0.0227 (19)
O3	0.0846 (17)	0.0428 (12)	0.116 (2)	0.0089 (11)	−0.0072 (15)	−0.0134 (13)
N1	0.0660 (16)	0.0560 (14)	0.0564 (15)	−0.0008 (12)	0.0121 (13)	0.0018 (11)
N2	0.0578 (15)	0.0609 (16)	0.0776 (19)	−0.0082 (12)	0.0063 (14)	0.0064 (14)
C1	0.072 (2)	0.0522 (17)	0.074 (2)	−0.0050 (15)	0.0258 (18)	−0.0059 (15)
C2	0.0563 (17)	0.0419 (15)	0.074 (2)	0.0001 (12)	0.0123 (16)	−0.0089 (13)
C3	0.0576 (19)	0.063 (2)	0.104 (3)	0.0012 (16)	0.023 (2)	−0.0148 (19)
C4	0.0562 (19)	0.060 (2)	0.121 (3)	0.0047 (16)	−0.005 (2)	−0.016 (2)
C5	0.066 (2)	0.065 (2)	0.097 (3)	0.0047 (17)	−0.013 (2)	−0.0094 (19)
C6	0.0626 (19)	0.0539 (17)	0.075 (2)	0.0047 (14)	0.0025 (16)	−0.0060 (15)
C7	0.0533 (16)	0.0406 (13)	0.0662 (19)	0.0012 (12)	0.0055 (14)	−0.0086 (12)
C8	0.0525 (16)	0.0470 (15)	0.0622 (18)	0.0033 (12)	0.0157 (14)	−0.0038 (13)
C9	0.0506 (15)	0.0482 (15)	0.0608 (17)	0.0027 (12)	0.0096 (14)	−0.0010 (13)
C10	0.0471 (15)	0.0548 (16)	0.0588 (17)	0.0004 (13)	−0.0032 (13)	0.0013 (13)
C11	0.072 (2)	0.0538 (18)	0.085 (2)	0.0046 (16)	0.0101 (18)	−0.0083 (16)
C12	0.091 (3)	0.061 (2)	0.100 (3)	0.008 (2)	0.000 (2)	−0.016 (2)
C13	0.101 (3)	0.053 (2)	0.111 (3)	−0.010 (2)	−0.020 (3)	0.000 (2)
C14	0.081 (2)	0.069 (2)	0.101 (3)	−0.020 (2)	0.001 (2)	0.013 (2)
C15	0.065 (2)	0.0516 (17)	0.067 (2)	0.0106 (15)	0.0176 (16)	−0.0061 (14)
C16	0.123 (4)	0.047 (2)	0.133 (4)	0.011 (2)	−0.006 (3)	−0.021 (2)
C17	0.099 (3)	0.092 (3)	0.065 (2)	−0.014 (2)	0.007 (2)	0.005 (2)
C18	0.116 (4)	0.134 (5)	0.104 (4)	0.021 (4)	−0.004 (3)	0.048 (3)
C19	0.154 (6)	0.099 (4)	0.140 (5)	0.034 (4)	0.026 (4)	0.021 (4)
C20	0.146 (6)	0.212 (9)	0.148 (6)	−0.029 (6)	0.012 (5)	−0.072 (6)
C22	0.106 (4)	0.139 (5)	0.117 (4)	0.042 (3)	0.010 (3)	0.034 (4)

### Geometric parameters (Å, °)

**Table:**

O1—C1	1.234 (5)	C21—C22	1.566 (9)
O2—C15	1.187 (4)	C3—H3	0.9300
O3—C15	1.324 (4)	C4—H4	0.9300
O3—C16	1.459 (5)	C5—H5	0.9300
N1—C1	1.372 (4)	C6—H6	0.9300
N1—C9	1.465 (4)	C8—H8	0.9800
N1—C17	1.461 (5)	C9—H9	0.9800
N2—C10	1.332 (4)	C11—H11	0.9300
N2—C14	1.313 (5)	C12—H12	0.9300
C1—C2	1.473 (5)	C13—H13	0.9300
C2—C3	1.413 (5)	C14—H14	0.9300
C2—C7	1.382 (5)	C16—H16A	0.9600
C3—C4	1.370 (6)	C16—H16B	0.9600
C4—C5	1.363 (6)	C16—H16C	0.9600
C5—C6	1.391 (5)	C17—H17A	0.9700
C6—C7	1.387 (5)	C17—H17B	0.9700
C7—C8	1.509 (4)	C18—H18A	0.9700
C8—C9	1.528 (4)	C18—H18B	0.9700
C8—C15	1.525 (4)	C19—H19A	0.9700
C9—C10	1.510 (4)	C19—H19B	0.9700
C10—C11	1.386 (5)	C20—H20A	0.9700
C11—C12	1.379 (6)	C20—H20B	0.9700
C12—C13	1.366 (7)	C21—H21A	0.9700
C13—C14	1.390 (7)	C21—H21B	0.9700
C17—C18	1.523 (8)	C22—H22A	0.9600
C18—C19	1.460 (9)	C22—H22B	0.9600
C19—C20	1.644 (11)	C22—H22C	0.9600
C20—C21	1.359 (10)		
			
O3···C6	3.294 (4)	H5···O3^v^	2.9000
O3···C2	3.292 (4)	H6···H8	2.4600
O1···H17A	2.3500	H6···O2^iv^	2.5400
O1···H3	2.5800	H8···N2	2.5700
O1···H22C^i^	2.8900	H8···H6	2.4600
O1···H12^ii^	2.7200	H9···O2	2.5200
O2···H16B	2.6100	H9···C18	2.7700
O2···H9	2.5200	H9···H17B	2.5800
O2···H14^iii^	2.8500	H9···H18A	2.2200
O2···H16C	2.6200	H11···N1	2.5800
O2···H6^iv^	2.5400	H11···C1	2.8400
O3···H5^v^	2.9000	H11···C17	3.0900
N1···H11	2.5800	H12···O1^i^	2.7200
N1···H19A	2.6500	H12···H19A^i^	2.5500
N2···H4^vi^	2.6600	H13···C3^i^	2.9800
N2···H8	2.5700	H14···O2^vii^	2.8500
C1···C11	3.386 (5)	H16B···O2	2.6100
C1···C15	3.498 (5)	H16C···O2	2.6200
C2···C10	3.431 (4)	H17A···O1	2.3500
C2···O3	3.292 (4)	H17A···H19B	2.4800
C5···C16^i^	3.534 (6)	H17A···H22A^ix^	2.5900
C5···C5^v^	3.366 (5)	H17B···C10	2.9800
C5···C6^v^	3.530 (5)	H17B···C11	3.0200
C6···O3	3.294 (4)	H17B···H9	2.5800
C6···C5^v^	3.530 (5)	H17B···C22^vii^	2.9900
C10···C2	3.431 (4)	H17B···H22B^vii^	2.5600
C11···C17	3.468 (6)	H18A···C9	2.8800
C11···C1	3.386 (5)	H18A···H9	2.2200
C15···C1	3.498 (5)	H18B···C21	3.0300
C16···C5^ii^	3.534 (6)	H18B···H21B	2.5200
C17···C11	3.468 (6)	H19A···N1	2.6500
C1···H19A	3.0700	H19A···C1	3.0700
C1···H11	2.8400	H19A···H12^ii^	2.5500
C3···H13^ii^	2.9800	H19B···H17A	2.4800
C9···H18A	2.8800	H19B···H21A	2.4700
C10···H22B^vii^	2.9700	H20A···H22C	2.3400
C10···H17B	2.9800	H20B···H22B	2.4100
C11···H17B	3.0200	H21A···H19B	2.4700
C17···H11	3.0900	H21B···C18	2.8700
C18···H9	2.7700	H21B···H18B	2.5200
C18···H21B	2.8700	H22A···H17A^x^	2.5900
C21···H18B	3.0300	H22B···H20B	2.4100
C22···H3^ii^	3.0900	H22B···C10^iii^	2.9700
C22···H17B^iii^	2.9900	H22B···H17B^iii^	2.5600
H3···O1	2.5800	H22C···H20A	2.3400
H3···C22^i^	3.0900	H22C···O1^ii^	2.8900
H3···H22C^i^	2.4500	H22C···H3^ii^	2.4500
H4···N2^viii^	2.6600		
			
C15—O3—C16	116.6 (3)	C15—C8—H8	108.00
C1—N1—C9	121.2 (3)	N1—C9—H9	107.00
C1—N1—C17	121.3 (3)	C8—C9—H9	107.00
C9—N1—C17	116.8 (3)	C10—C9—H9	107.00
C10—N2—C14	118.8 (3)	C10—C11—H11	120.00
O1—C1—N1	121.2 (3)	C12—C11—H11	120.00
O1—C1—C2	121.8 (3)	C11—C12—H12	120.00
N1—C1—C2	117.0 (3)	C13—C12—H12	120.00
C1—C2—C3	119.7 (3)	C12—C13—H13	122.00
C1—C2—C7	121.6 (3)	C14—C13—H13	122.00
C3—C2—C7	118.7 (3)	N2—C14—H14	118.00
C2—C3—C4	120.5 (4)	C13—C14—H14	118.00
C3—C4—C5	120.6 (3)	O3—C16—H16A	109.00
C4—C5—C6	119.9 (4)	O3—C16—H16B	109.00
C5—C6—C7	120.4 (3)	O3—C16—H16C	110.00
C2—C7—C6	119.9 (3)	H16A—C16—H16B	109.00
C2—C7—C8	118.0 (3)	H16A—C16—H16C	109.00
C6—C7—C8	122.2 (3)	H16B—C16—H16C	109.00
C7—C8—C9	110.3 (2)	N1—C17—H17A	109.00
C7—C8—C15	114.7 (2)	N1—C17—H17B	109.00
C9—C8—C15	109.0 (2)	C18—C17—H17A	109.00
N1—C9—C8	110.4 (2)	C18—C17—H17B	109.00
N1—C9—C10	114.1 (2)	H17A—C17—H17B	108.00
C8—C9—C10	111.3 (3)	C17—C18—H18A	109.00
N2—C10—C9	114.6 (3)	C17—C18—H18B	109.00
N2—C10—C11	121.2 (3)	C19—C18—H18A	109.00
C9—C10—C11	124.1 (3)	C19—C18—H18B	109.00
C10—C11—C12	119.1 (3)	H18A—C18—H18B	108.00
C11—C12—C13	120.0 (4)	C18—C19—H19A	106.00
C12—C13—C14	116.8 (4)	C18—C19—H19B	106.00
N2—C14—C13	124.2 (4)	C20—C19—H19A	106.00
O2—C15—O3	123.1 (3)	C20—C19—H19B	106.00
O2—C15—C8	123.5 (3)	H19A—C19—H19B	106.00
O3—C15—C8	113.4 (3)	C19—C20—H20A	110.00
N1—C17—C18	114.5 (4)	C19—C20—H20B	110.00
C17—C18—C19	113.9 (5)	C21—C20—H20A	110.00
C18—C19—C20	125.5 (6)	C21—C20—H20B	110.00
C19—C20—C21	110.1 (7)	H20A—C20—H20B	108.00
C20—C21—C22	108.6 (6)	C20—C21—H21A	110.00
C2—C3—H3	120.00	C20—C21—H21B	110.00
C4—C3—H3	120.00	C22—C21—H21A	110.00
C3—C4—H4	120.00	C22—C21—H21B	110.00
C5—C4—H4	120.00	H21A—C21—H21B	108.00
C4—C5—H5	120.00	C21—C22—H22A	110.00
C6—C5—H5	120.00	C21—C22—H22B	109.00
C5—C6—H6	120.00	C21—C22—H22C	109.00
C7—C6—H6	120.00	H22A—C22—H22B	109.00
C7—C8—H8	108.00	H22A—C22—H22C	109.00
C9—C8—H8	107.00	H22B—C22—H22C	109.00
			
C16—O3—C15—O2	−0.7 (6)	C5—C6—C7—C8	179.5 (3)
C16—O3—C15—C8	178.6 (3)	C5—C6—C7—C2	0.3 (5)
C9—N1—C1—O1	−174.2 (3)	C6—C7—C8—C15	−89.5 (4)
C17—N1—C1—C2	175.6 (3)	C2—C7—C8—C9	−33.9 (4)
C9—N1—C17—C18	−72.9 (4)	C6—C7—C8—C9	146.9 (3)
C17—N1—C9—C10	−84.3 (3)	C2—C7—C8—C15	89.7 (3)
C17—N1—C1—O1	−4.1 (5)	C7—C8—C9—N1	52.2 (3)
C9—N1—C1—C2	5.6 (4)	C9—C8—C15—O2	−54.3 (5)
C1—N1—C17—C18	116.7 (4)	C9—C8—C15—O3	126.4 (3)
C1—N1—C9—C10	86.2 (3)	C15—C8—C9—C10	157.7 (2)
C1—N1—C9—C8	−40.0 (4)	C7—C8—C15—O2	−178.5 (4)
C17—N1—C9—C8	149.5 (3)	C7—C8—C15—O3	2.2 (4)
C14—N2—C10—C11	1.1 (5)	C7—C8—C9—C10	−75.5 (3)
C14—N2—C10—C9	−175.4 (3)	C15—C8—C9—N1	−74.6 (3)
C10—N2—C14—C13	−1.9 (6)	N1—C9—C10—C11	−7.2 (4)
N1—C1—C2—C7	16.6 (4)	C8—C9—C10—N2	−65.2 (3)
O1—C1—C2—C3	14.9 (5)	C8—C9—C10—C11	118.5 (3)
N1—C1—C2—C3	−164.9 (3)	N1—C9—C10—N2	169.1 (3)
O1—C1—C2—C7	−163.6 (3)	N2—C10—C11—C12	−0.3 (5)
C3—C2—C7—C8	−179.1 (3)	C9—C10—C11—C12	175.9 (3)
C3—C2—C7—C6	0.1 (4)	C10—C11—C12—C13	0.2 (6)
C1—C2—C7—C6	178.7 (3)	C11—C12—C13—C14	−0.8 (7)
C1—C2—C7—C8	−0.5 (4)	C12—C13—C14—N2	1.8 (7)
C7—C2—C3—C4	−0.3 (5)	N1—C17—C18—C19	−66.1 (6)
C1—C2—C3—C4	−178.9 (3)	C17—C18—C19—C20	171.8 (6)
C2—C3—C4—C5	0.1 (5)	C18—C19—C20—C21	60.3 (10)
C3—C4—C5—C6	0.4 (5)	C19—C20—C21—C22	−177.8 (5)
C4—C5—C6—C7	−0.6 (5)		

Symmetry codes: (i) −*x*+3/2, *y*+1/2, *z*; (ii) −*x*+3/2, *y*−1/2, *z*; (iii) −*x*+5/2, *y*−1/2, *z*; (iv) −*x*+2, −*y*+2, −*z*+1; (v) −*x*+1, −*y*+2, −*z*+1; (vi) *x*+1, *y*, *z*; (vii) −*x*+5/2, *y*+1/2, *z*; (viii) *x*−1, *y*, *z*; (ix) −*x*+2, *y*+1/2, −*z*+3/2; (x) −*x*+2, *y*−1/2, −*z*+3/2.

### Hydrogen-bond geometry (Å, °)

**Table:**

*D*—H···*A*	*D*—H	H···*A*	*D*···*A*	*D*—H···*A*
C6—H6···O2^iv^	0.93	2.54	3.460 (5)	169
C8—H8···N2	0.98	2.57	2.983 (4)	105
C9—H9···O2	0.98	2.52	2.928 (4)	105
C11—H11···N1	0.93	2.58	2.896 (4)	100
C17—H17A···O1	0.97	2.35	2.778 (5)	106

Symmetry codes: (iv) −*x*+2, −*y*+2, −*z*+1.
